# Static and Dynamic Adaptation of Insect Photoreceptor Responses to Naturalistic Stimuli

**DOI:** 10.3389/fphys.2016.00477

**Published:** 2016-10-25

**Authors:** Andrew S. French, Esa-Ville Immonen, Roman V. Frolov

**Affiliations:** ^1^Department of Physiology and Biophysics, Dalhousie UniversityNova Scotia, CA, Canada; ^2^Department of Physics, University of OuluOulu, Finland

**Keywords:** log-normal distribution, nonlinear dynamics, block-structured, naturalistic stimuli, phototransduction, noise, entropy

## Abstract

We describe a new nonlinear dynamic model of insect phototransduction using a NLN (nonlinear, linear, nonlinear) block structure. The first nonlinear stage provides a single exponential decline in gain and mean following the start of light stimulation. The linear stage uses a two-parameter log-normal convolution model previously applied alone to insect photoreceptors. The final stage is a static quadratic function. The model fitted current and voltage responses of isolated single photoreceptors from three different insect species with reasonable fidelity when they were stimulated by naturalistic time series having wide bandwidth and contrast, over a light intensity range of >1:10^4^. Mean squared error values for receptor current and receptor potential varied over ~2–60%, with many values below 10%. Linear log-normal filter parameters did not vary strongly with species or light intensity. Initial gain reduction was only large for the highest light levels, while the time constant of gain and mean reduction decreased with light intensity. The final nonlinearity changed from positively to negatively quadratic with increasing light intensity, indicating a change from threshold, or expansion to saturating compression with greater signal strength. Photoreceptor information transmission was estimated by linear information capacity and signal entropy measurements of both experimental data and predicted outputs of the model for identical stimuli at each light level. Comparison of actual and predicted data indicated significant added noise during phototransduction, with information being progressively lost by nonlinear behavior with increasing light intensity.

## Introduction

Dynamic responses of vertebrate and invertebrate photoreceptors are difficult to explain, either by analytical descriptions or by photochemical reaction cascades. A single flash of light produces a delayed, transient change in membrane current that is a nonlinear function of flash intensity and background illumination (Hartline and McDonald, 1947; Fuortes and Hodgkin, 1964). Existing molecular models of insect phototransduction cannot account for these system dynamics, at least partially because the mechanisms that open ion channels to create the receptor current are still unclear (Hardie and Juusola, 2015).

An analytical model comprising a cascade of simple linear filters was used to explain the time course of single flash responses in the *Limulus* eye, particularly the delay between the flash and the initial rise in current (Fuortes and Hodgkin, 1964). Although such filters could plausibly be explained by simple chemical reactions (Borsellino et al., 1965), the number of filters required was so large (often exceeding 10), that the model seemed unrealistic. One alternative was to incorporate a fixed delay, of unknown mechanism, which allowed a simpler linear filter model with a smaller number of parameters to explain the remaining response to both flashes and randomly fluctuating light signals (French, 1980). Another suggestion was to convolve the light signal with a nonlinear function of time, the log-normal function, which using only two parameters could account for the delayed response in a range of insect photoreceptor responses (Payne and Howard, 1981; Howard et al., 1984), including single photon responses (Henderson et al., 2000).

Although linear convolution with a filter function provided a close description to single flash responses and random fluctuations around a mean light intensity, insect photoreceptors clearly demonstrate nonlinear adaptation, even under asymptotically small signal conditions (Marmarelis and McCann, 1977; Laughlin and Hardie, 1978; Pece and French, 1992). Nonlinear analyses of flash responses and frequency responses suggested that the processes between light absorption and membrane conductance change included both an early gain reduction and a late saturation with light intensity (Weckström et al., 1988; Pece et al., 1990; French et al., 1993). Known sources of nonlinearity include electrical shunting by ion channels in the cell membrane (Weckström et al., 1988, 1995), dynamic changes in the size, shape, and latency of quantum bumps (Song et al., 2012), and blockage or depletion of Ca^2+^ entry through light-activated channels (Hardie and Mojet, 1995; Chu et al., 2013b). Additionally, a range of interactions between voltage-activated channels and the transduced light current are now well-established (Weckström and Laughlin, 1995; Juusola et al., 2003; Niven et al., 2004).

Photoreceptors are inherently noisy transducers because of the stochastic distribution of photon arrival, but additional sources of noise include variability in the transduction cascade and stochastic properties of membrane ion channels (Barlow, 1956; Wu and Pak, 1978; Lillywhite and Laughlin, 1979; Laughlin and Lillywhite, 1982; Henderson et al., 2000; Chu et al., 2013a). Both noise and nonlinearity can cause a loss of information as the light signal is transduced, but initial attempts to quantify such losses concentrated on signal-to-noise ratios estimated from linear models of transduction (Bendat and Piersol, 1980; Kouvalainen et al., 1994; Niven et al., 2003). More recent work has considered nonlinear effects on sensory information transmission in several sensory receptors, using naturalistic stimuli that approximate the natural range of amplitude distributions and dynamics (van der Schaaf and van Hateren, 1996; Juusola and de Polavieja, 2003; Niven et al., 2004). Accompanying this development has been a change of emphasis from communication channel information capacity (Shannon and Weaver, 1949) to nonlinear measurements of signal information based on entropy, as estimated from probability distributions (Juusola and de Polavieja, 2003; Takalo et al., 2011) or by data compression (Pfeiffer and French, 2009).

In the present study, we developed a new nonlinear model of phototransduction based on an extension of the log-normal method (Payne and Howard, 1981) to include early gain adaptation and a final nonlinearity. The model combines log-normal convolution with the nonlinear-linear-nonlinear cascade structure developed previously for several sensory systems, including phototransduction (Marmarelis and Marmarelis, 1978; Weckström et al., 1988; French et al., 1993). Model construction was also guided by evidence of early gain changes in insect photoreceptors (Pece et al., 1990; Friederich et al., 2012). The final nonlinearity employed a polynomial series, for generality, and as used previously for insect photoreceptors (French et al., 1993). We fitted the model to photoreceptor membrane potential and membrane current recordings produced by naturalistic light fluctuations from three different types of insects that operate in widely varying visual environments. We required the model to account for the transient adaptation at the start of light stimulation from a dark background, as well as the static adaptation represented by changes in dynamic response to different mean light intensity stimuli.

The model was able to reproduce responses to naturalistic stimulation of 60 s duration, starting from dark, and over a range of more than 1:10,000 in stimulus amplitude, with mean squared error between model and fitted data as low as 2%. Initial gain adaptation was strongest and fastest under the brightest conditions, but the two parameters of the log-normal component did not change strongly with species or light intensity. The final nonlinearity, approximated by a second-order polynomial function, changed from positively to negatively quadratic with light intensity, indicating an appropriate adaptation to available signal strength. Although linear coherence (signal-to-noise) suggested relatively poor information transfer during transduction under all conditions, we found that most of the input signal entropy was actually recovered by the nonlinear models at the lowest illumination levels.

## Materials and methods

### Animals, stimulation, and recording

All experiments were conducted in accordance with EU Directive 2010/63/EU for animal experiments. Cockroaches, *Periplaneta americana*, and crickets, *Gryllus bimaculatus*, were obtained from Blades Biological Ltd. (Edenbridge, Kent, UK) and maintained at 25°C under inverse 12–12 h illumination conditions, with experiments performed on dark-adapted insects during daytime. Adult backswimmers (*Notonecta glauca*) were collected locally in Oulu (Finland) or purchased from Blades Biological Ltd. Photoreceptors were always allowed to adapt to dark conditions for periods of several minutes before recordings. Some recordings from *N. glauca* and *G. bimaculatus* were used previously (Frolov and Weckström, 2014; Immonen et al., 2014a). Ommatidia were dissociated as described previously (Krause et al., 2008; Immonen et al., 2014b). Whole-cell recordings from dissociated ommatidia were performed at room temperature (20–22°C) as described previously (Hardie et al., 1991; Krause et al., 2008). In brief, an Axopatch 1-D patch-clamp amplifier and pClamp 9.2 software (Axon Instruments/Molecular Devices, CA, USA) were used for data acquisition and analysis. Patch electrodes were fabricated from thin-walled borosilicate glass (World Precision Instruments, Sarasota, FL, USA). Electrodes had a resistance of 5–15 MΩ. Bath solution contained (in mM): 120 NaCl, 5 KCl, 4 MgCl_2_, 1.5 CaCl_2_, 10 N-Tris-(hydroxymethyl)-methyl-2-amino-ethanesulfoncic acid (TES), 25 proline and 5 alanine, pH 7.15. Patch pipette solution contained (in mM): 140 KCl, 10 TES, 2 MgCl_2_, 4 Mg-ATP, 0.4 Na-GTP, and 1 NAD, pH 7.15. All chemicals were purchased from Sigma Aldrich Inc. (St. Louis, USA). The liquid junction potential (LJP) between bath and intracellular solution was −4 mV. A holding potential of −74 mV (including LJP) was used for voltage-clamp recordings. The series resistance was compensated by at least 80%, with access resistance after compensation typically not exceeding 15 MΩ. Recordings were performed from green-sensitive photoreceptors. A 60 s naturalistic contrast sequence from the van Hateren natural image database was used as the input signal to drive the light stimulus (van der Schaaf and van Hateren, 1996).

### Data analysis

Membrane current and membrane potential were initially sampled at a rate of 1200 Hz (0.833 ms sample interval). Preliminary measurements found negligible power in the input or output signals above 50 Hz, so all data files were ten-point averaged to give a resolution of 8.33 ms.

Coherence functions, γ^2^(*f*), where *f* is frequency, for each input-output set were obtained from the spectra of the input, *S*_xx_(*f*), output, *S*_yy_(*f*), and cross-spectra, *S*_xy_(*f*) (Bendat and Piersol, 1980):

(1) $$\gamma^{2}(f)=\;<\;|S_{xy}(f){|}^{2}>/(<S_{xx}(f)><S_{yy}(f)>)$$

where < > indicate ensemble averages. Linear information capacity, *R*, was estimated from (Juusola and French, 1997):

(2) $$R=\int log_{2}(1/(1-\gamma^{2}(f)))df$$

Signal entropy was estimated as described previously (Pfeiffer et al., 2012). Signals were normalized and digitized so that the maximum amplitude range could be represented by 10-bit numbers or 1024 different amplitude levels. Entropy was obtained by context-independent data compression of regularly sampled continuous signals. Each of the 1024 numerical values representing the digitized signal was treated as an independent symbol in a linear sequence, or message. Data compression was performed by repeatedly replacing pairs of symbols that occurred with greatest frequency by new symbols, until no further compression was achieved. The entropy, *E*, was then given by:

(3) $$E=(N\;log_{2}M)/10$$

where *N* is number of symbols in the compressed message and *M* is the number of different symbols in the message and the division by 10 compensates for digitization (Cover and Thomas, 1991).

### Photoreceptor model

**Figure 1:**
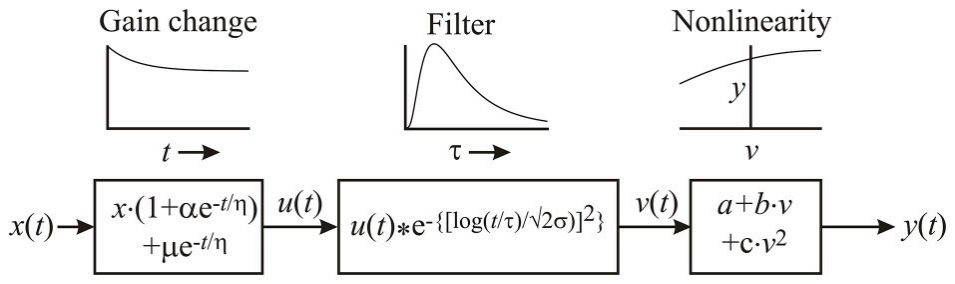
**Parametric model of transduction in insect photoreceptors**. Light fluctuation as a function of time, *x*(*t*), passes through an initial stage that reduces its amplitude, and changes its mean level by an exponentially decaying function of time after initial stimulation. Three parameters define this stage: α, the total proportional change in amplitude; μ, the total change in mean level; and η, the time constant for both. The resulting signal, *u*(*t*), is convolved with the log-normal photoreceptor filter function of Payne and Howard (1981), with its two parameters, τ and σ. Finally, the output of the log-normal filter, *v*(*t*) passes through a static (time independent) nonlinearity formed by a second-order polynomial function with parameters *a, b*, and *c*.

The same model system (Figure 1) was used to simulate both photoreceptor current and potential. The model was based on the log-normal model of Payne and Howard (1981), shown in the center box of Figure 1, but preceded by a nonlinear component that reduces the overall gain of the system with time from the start of stimulation by including an additional amplitude parameter, α, whose effect declines exponentially with time constant, η. Since the initial gain change was usually accompanied by a small change in mean current or potential, we included an addition to the mean, μ, that decays by the same time constant, η. The final stage of the model consists of a static (memory-less) nonlinear change in amplitude and mean approximated by a second order polynomial function with parameters *a, b*, and *c* (Figure 1). The overall gain of the model, including conversion from light intensity to membrane potential or current is assumed to occur in the final stage, but the polynomial displays were normalized to unit input and output for graphical display.

Fitting the model to the data was performed on 9000 input-output data pairs by simulated annealing (Kirkpatrick et al., 1983; Press et al., 1990), brute force and Levenberg–Marquardt (Marquardt, 1963) methods to minimize the mean square error (MSE) between receptor current or receptor potential output, *y*(*t*), and the simulated output, *y*_s_(*t*):

(4) $$\text{MSE}=100[{\left(y(t)-y_{\text{s}}(t)\right)}^{2}]/([y^{2}(t)]-{\left[y(t)\right]}^{2})$$

where [] indicate time averages (French and Marmarelis, 1999). All software for model fitting, entropy and information capacity estimation was custom written in multi-threaded C++ and operated on standard desktop personal computers.

## Results

Experiments were performed on six dissociated receptor cells from *Periplaneta*, plus single cells from *Gryllus* and *Notonecta*. The naturalistic stimulus sequence was from a collection obtained by an animal (human) moving forward through a natural visual environment under controlled conditions of motion and light detection (van der Schaaf and van Hateren, 1996). Each cell was stimulated with the same naturalistic stimulus sequence a total of 10 times, recording receptor current and receptor potential with five different neutral density filters (ND) in the light path. Each recording started from the dark adapted state, so the maximum contrast (brightest light to dark) increased by a factor of 10 for each ND change. Actual light levels were estimated by counting single photon arrivals as current bumps (effective photons) under the darkest stimulation conditions during the 60 s stimulation. These values were then scaled by the appropriate number of ND filters in each experiment. Some recordings were lost before the set of experiments were complete, so from a total possible of 80 recordings (10 recording from each of eight cells) a total of 47 recordings were obtained (25 receptor potential and 22 receptor current). Mean values of fitted parameters were calculated for the *Periplaneta* data, but standard deviations are only shown when there were at least three measurements.

Each experiment required fitting the eight parameters of the model (Figure 1) to 9000 input-output pairs. We used primarily the simulated annealing approach (Kirkpatrick et al., 1983; Press et al., 1990) for parameter fitting, but each fitting was also tested by brute force and Levenberg–Marquardt methods (Marquardt, 1963) numerous times during the fitting process. We also used different starting parameter values several times to test for convergence in each case. These constraints required periods of hours (sometimes overnight) for each fitting. Note, that error (MSE) values were based on the entire data record during each fitting process because the non-stationary nature of the data and model, combined with the limited data available, prevented validation on separate experimental records.

### Initial gain reduction

**Figure 2:**
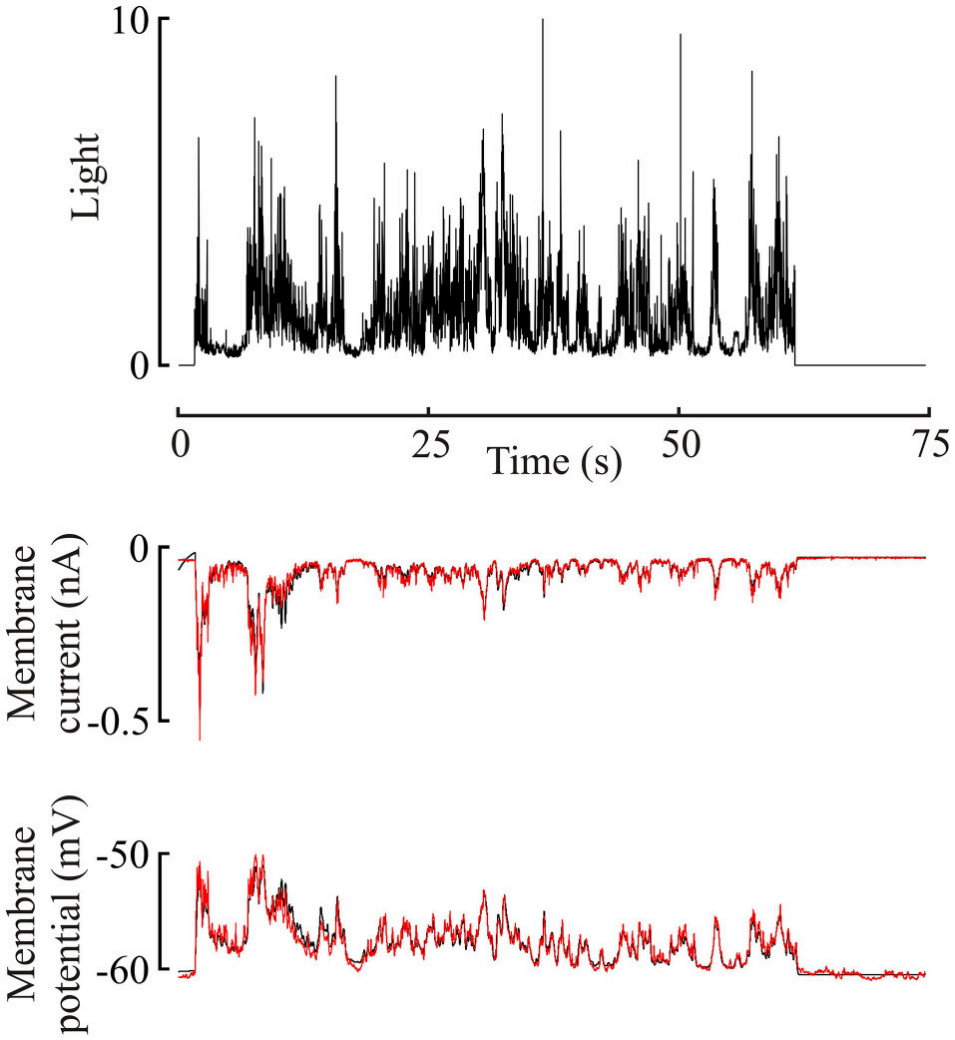
**Membrane current and potential changes in a *Periplaneta* photoreceptor during 60 s naturalistic light stimulation (van der Schaaf and van Hateren, 1996)**. Light stimulus in the upper trace, with membrane current and potential in the middle and lower traces, respectively. This light level gave an estimated mean response of 620 ep/s. Experimental current and potential (black) are plotted with superimposed responses from the model of Figure 1 to the same stimulus (red), using the best-fitting parameters for this data. Note, that model data reproduces experimental data well-enough to obscure most of the underlying (black) plot.

**Figure 3:**
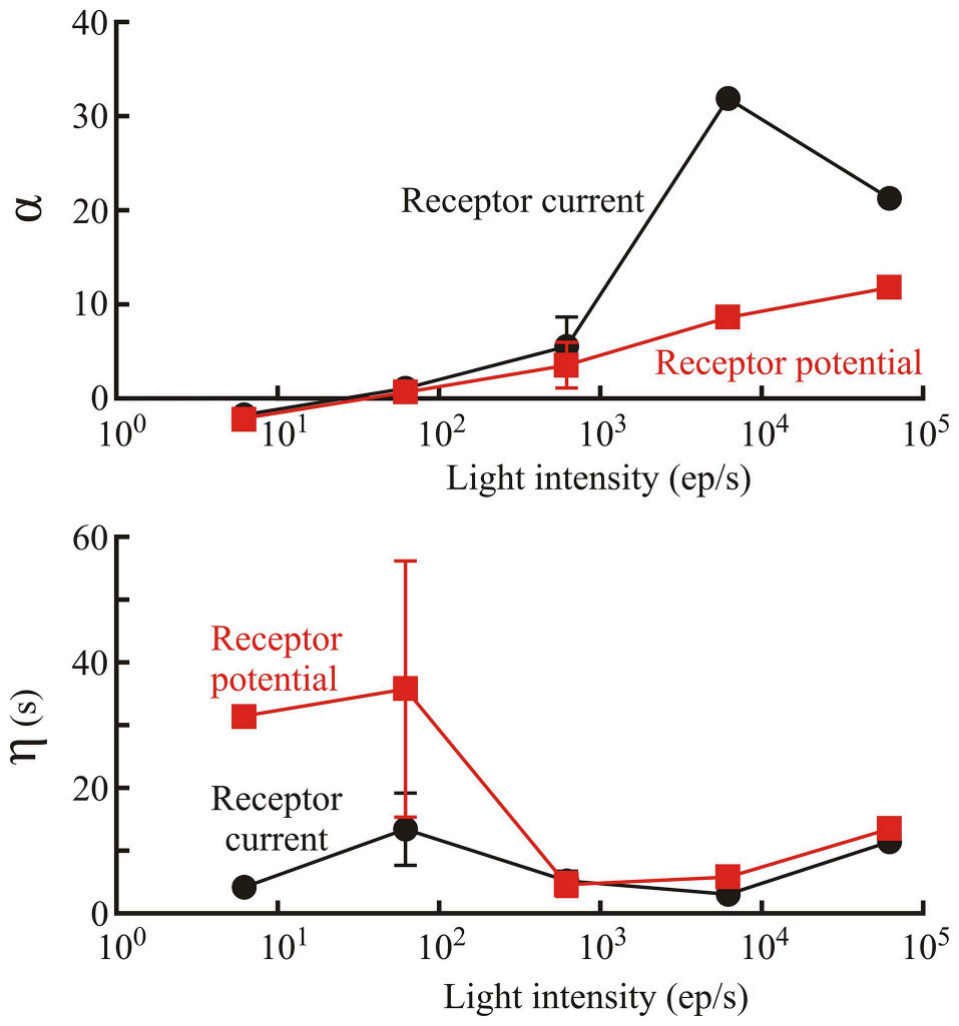
**Parameters defining the initial gain change of the phototransduction model (first box of Figure 1)**. Amplitude, α; and time constant, η; of gain change are shown as functions of light level, estimated from photon counts, in *Periplaneta* photoreceptors. Numbers of experiments contributing to each data value were: 1, 6, 5, 2, and 1 for increasing light levels. Mean values of multiple experiments are shown, and standard deviations are shown for experiments with five and six estimates. Note, that α is dimensionless because the conversion to current or potential was considered to occur in the final nonlinear stage of the model.

Membrane current and membrane potential changes during the 60 s of naturalistic light stimulation could be fitted by the model (Figure 2), even at the earliest stimulation times when the gain of the photoreceptors was clearly decreasing. This is an important feature of the model. Error (MSE, Equation 4) values at the completion of fitting ranged from 2.1 to 62.9%, with 16 of the 47 MSE values at or below 10%. MSE values were always higher for receptor current than receptor potential, and error levels were similar for all three species. The highest error values were only observed under the dimmest light conditions. Gain changes after the start of light stimulation (first component of Figure 1) were larger (amplitude parameter α) and more rapid (time constant parameter η) at higher maximum light intensities (Figure 3). These effects were seen in both membrane current and potential recordings, and the fitted gain change parameters agreed for the two types of recordings.

### Log-normal filter

**Figure 4:**
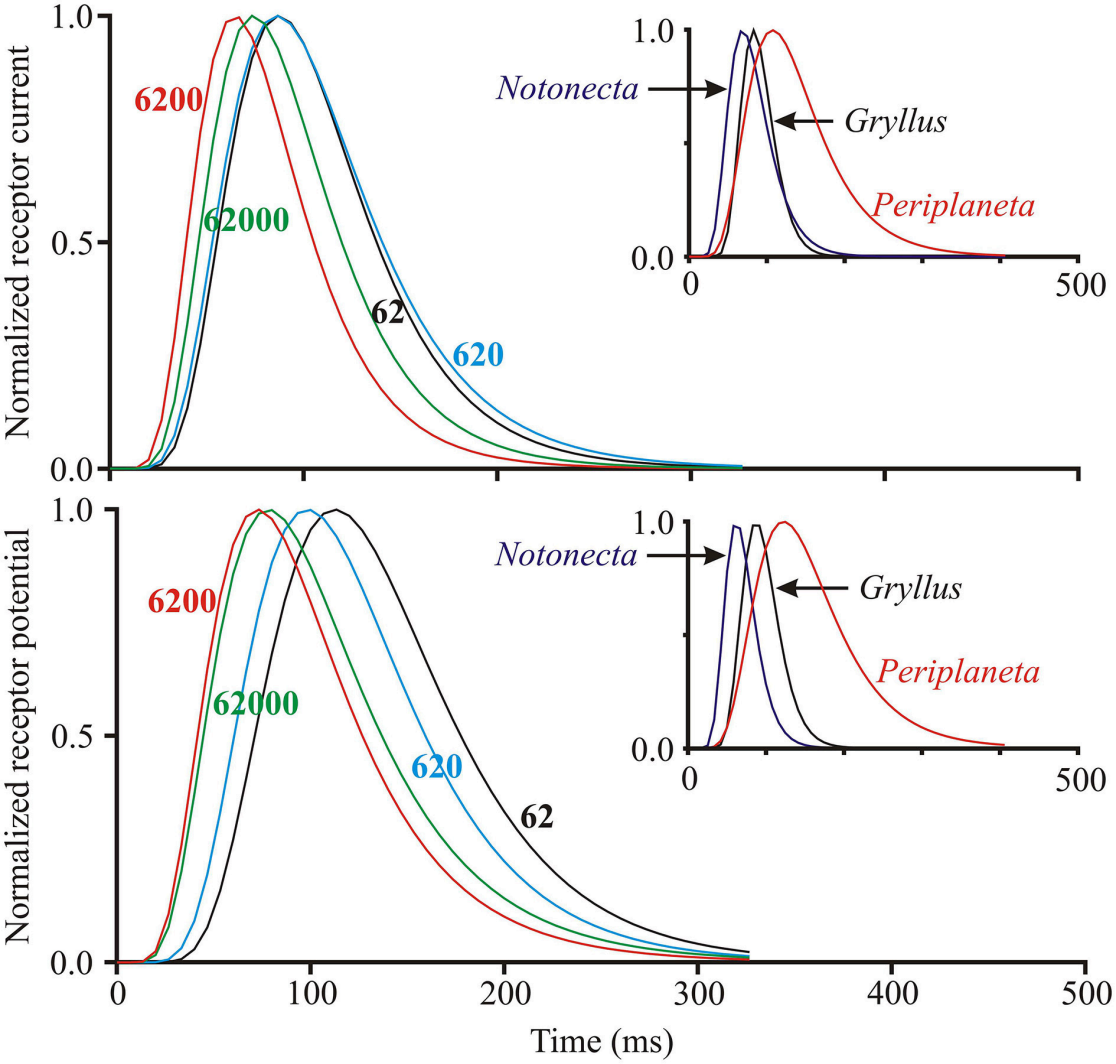
**Log-normal filter function of the insect photoreceptor model (Figure 1) for varying light intensities and species**. Main plots show curves generated from fitted parameters for both receptor current and receptor potential at four different levels of mean light stimulus (values next to curves in ep/s) in a single photoreceptor cell from *Periplaneta*. Curves moved to the right at lower light levels, corresponding to slower responses. Insets show similar curves at intermediate levels for three species with different dynamic responses: *Notonecta* (200 ep/s), *Gryllus* (560 ep/s), and *Periplaneta* (620 ep/s).

**Figure 5:**
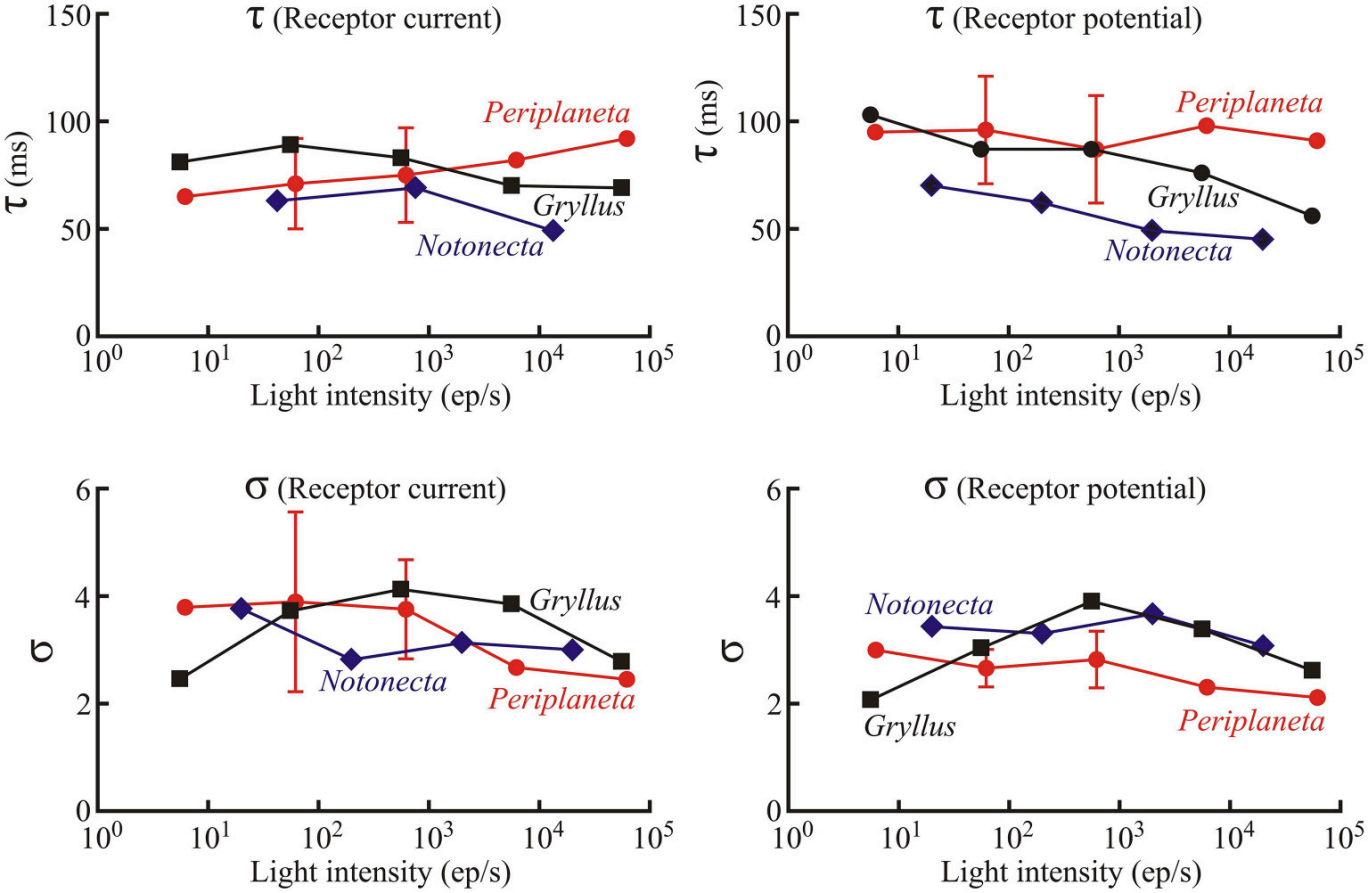
**Values of the Log-normal parameters, τ and σ, as functions of light intensity as in Figure 3**. Numbers of experiments contributing to *Periplaneta* data were: 1, 6, 5, 2, and 1 for increasing light levels. Mean values of multiple experiments are shown, and standard deviations are shown for experiments with five and six estimates. All other values represent fitted values to single experiments.

In contrast to the initial gain changes, fitted parameters of the log-normal filter (center component of Figure 1, time constant τ and width parameter σ) did not vary strongly with light level or with species (Figures 4, 5). As a *Periplaneta* example shows (Figure 4), the peak response shifted by less than a factor of two over the light intensity range of 1:1000. The log-normal filter parameters varied with the different species used, being most rapid for *Notonecta* and slowest for *Periplaneta* (Figure 4 insets). Mean parameter values (τ and σ) for receptor potential models were approximately constant at different light levels (Figure 5). Mean parameters for receptor current showed some slowing and broadening of the response at the lowest light levels, but there were not enough data to test for statistical significance. The smaller sets of data for *Notonecta* and *Gryllus* agreed well with the mean *Periplaneta* data, but again showed faster responses, especially for *Notonecta*, and more clearly at higher light levels.

### Output nonlinearity

**Figure 6:**
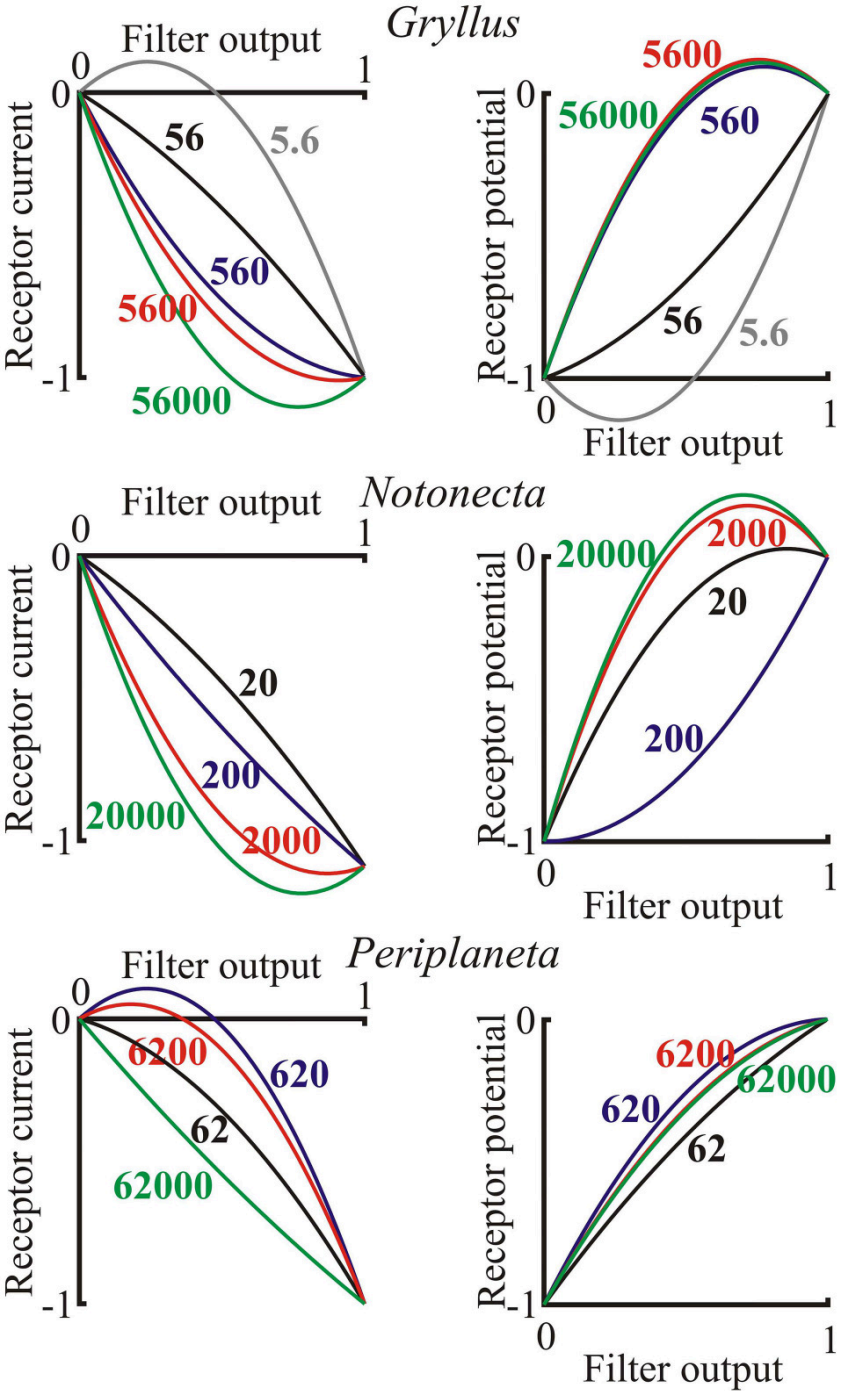
**Nonlinear functions representing the final stage of the photoreceptor model (Figure 1) for both receptor current and receptor potential**. Axes were normalized to the output range of the filter function, as input, and the final current or potential range as output. Data are shown for single examples of the three species as functions of the input light intensity in effective photons per second, indicated on each curve. Note, that these are only second-order polynomials, so output values exceeding the inputs in some cases are only approximations to the final nonlinearity, which would probably be reduced by higher order terms.

The final static nonlinearity was modeled by a second-order polynomial function of the output from the log-normal filter (last component of Figure 1). Nonlinear functions are shown for the three species over the range of light levels, but with the full ranges of the input and output signals to each function normalized to unity, in order to show the effects of the nonlinearities (Figure 6). There was a clear general transition from positive, expansive functions at low light intensities to saturating, compressive functions as light intensity increased in both current and potential for all species. Negative and positive overshoots of the functions were presumably caused by the limited number of polynomial terms in the estimates, suggesting that the responses tend to exhibit threshold behavior at the lowest light intensities and strong saturation at the highest intensities.

### Information capacity, transfer, and entropy

**Figure 7:**
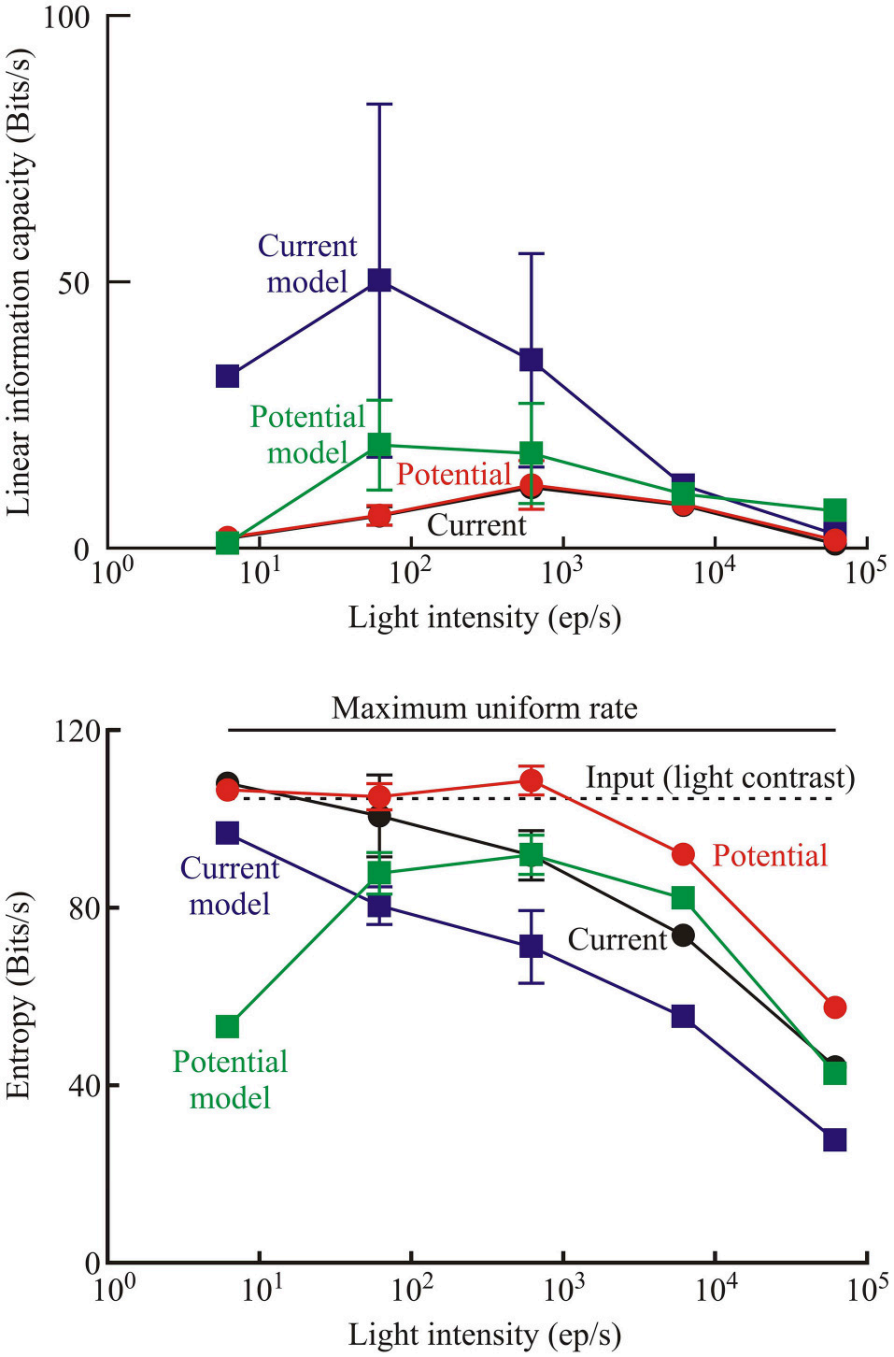
**Measures of information transmission by photoreceptors transducing naturalistic stimulation**. Upper: linear information capacity calculated from the coherence function between the input and output data for receptor current, receptor potential, and the respective models of current and potential for the *Periplaneta* receptors as a function of input light intensity. Lower: entropy rates in the photoreceptors measured by data compression for the same signals in the *Periplaneta* receptors. Numbers of experiments were: 1, 6, 5, 2, and 1 for increasing light levels. Mean values of multiple experiments are shown, and standard deviations are shown for experiments with five and six estimates. All other values represent fitted values to single experiments. Dashed line indicates the entropy rate of the input light signal. Solid upper line shows the maximum entropy rate that could be calculated by this method, corresponding to a uniform distribution of values over the same range.

The photoreceptor models did not add uncorrelated or correlated noise to the transduced signal, which allowed some separation of the relative contributions of noise and nonlinearity to limiting information transmission by the experimental photoreceptors. Information capacity between input naturalistic light stimulus and output membrane current and membrane potential were estimated from the coherence function (Equation 3). Similar measurements were then made by feeding the same input sequence into the best-fitting model (Figure 1) for each recording. Mean values of these data are shown for the different light intensities used in the *Periplaneta* experiments (Figure 7, upper). Total signal entropy of the input time sequence, resulting membrane potential, membrane current, and corresponding model outputs were measured by data compression (Pfeiffer et al., 2012). Mean values of these data are also shown for the different intensities in the *Periplaneta* experiments (Figure 7, lower).

Information capacities were low for the experimental data, both for membrane potential and current, with no definite trend vs. light intensity. The fitted models gave higher values, particularly at low intensities. Information capacity can be reduced by uncorrelated noise or by nonlinearity, but the models were purely parametric and did not add uncorrelated noise. Since a linear, noise-free system has infinite information capacity, it follows that the reduced capacity of the models was entirely due to nonlinearity.

Signal entropy also increased at lower light intensities, for both experimental data and modeled responses, and approached the constant value for the input signal entropy in some cases. Input entropy was close to, but below the maximum theoretical entropy that could be produced by this estimation technique (dashed line, Figure 7), indicating that the naturalistic signal exercised the receptors over their full response ranges.

## Discussion

The wide dynamic and intensity ranges of natural light stimulation require nonlinear compression and adaptation processes to avoid saturation and allow adequate signal-to-noise levels in the photoreceptor membrane potential fluctuations (Laughlin and Hardie, 1978; van Hateren, 1997; van Hateren and Snippe, 2001). Relatively simple linear (French, 1980) and nonlinear (French et al., 1993) models give reasonable simulation of controlled inputs such as white Gaussian noise and steps, but the present results show that several interacting nonlinear and linear processes may be necessary to explain complete photoreceptor transduction function.

Although the log-normal model has been available for decades (Payne and Howard, 1981) this work describes the first application of the model to naturalistic data. Gain change in the early stages of insect eye transduction models has been described previously (Pece et al., 1990; Friederich et al., 2012), and is clearly justified by the form of the responses (Figure 2). Simple exponential reduction in gain provided good agreement with the experimental data, including the strong amplitude changes at the start of stimulation. Gain change was much stronger and faster at the highest light levels (Figure 4). More complex forms of initial nonlinearity have been suggested for insect phototransduction before, including changing dynamics in *Locusta* (Pece et al., 1990) and multiple time constants of change in *Locusta* (Laughlin and Hardie, 1978) and *Drosophila* (Friederich et al., 2012) but it would be difficult to justify the addition of more fitting parameters for the present *Periplaneta* data.

The model fitted both membrane current and membrane potential. Receptor current fluctuations cause receptor potential fluctuations via the membrane time constant plus any other ionic currents induced by the potential changes. Typical membrane time constants are much smaller than the time scales of the model (Figures 3, 4), and while parameter differences between current and potential, such as the log-normal fits, may reflect receptor physiology, there are not enough data to make statistically valid arguments. Error values were generally higher for current than potential fitting, which may reflect filtering of inherent noise by the membrane or different experimental noise.

### Fitted parameters

Suggested mechanisms of gain change in insect photoreceptors include optical phenomena, such as changes in the acceptance angle due to rhabdomere or screening pigment migration (Immonen et al., 2014a), changes in the phototransduction cascade itself, and membrane electrochemistry, particularly shunting (Laughlin, 1989). The time course of the gain change that we observed (up to 30 s—Figure 3) suggests a relatively slow process like pigment migration rather than more rapid membrane phenomena.

Although the log-normal filter became faster at brighter levels (Figures 4, 5) these changes were not large, and might not even be statistically significant if more data were available. This relative refractoriness may reflect the wide dynamic and amplitude ranges of the naturalistic stimulus; since the model was required to fit the responses over the whole period of stimulation, and may therefore represent an average description of the photoreceptor dynamics over these wide stimulation ranges.

In contrast to the slow initial gain change, the nonlinear function at the end of the model cascade was static. While some responses were approximately linear, we observed both expansive and compressive behavior as the light intensity increased. The apparent expansion may represent some form of threshold behavior at low light levels. Compressive saturation of electrical responses is well-known in insect photoreceptors, with at least one mechanism being the shunting of transduction current by voltage activated ion channels as the cell depolarizes (Weckström et al., 1988, 1995; French et al., 1993). Saturating nonlinearities in receptor current at higher light intensities (Figure 6), suggest that some nonlinearities occur before ion channels are opened. However, current data must always be treated with caution because of the difficulties of achieving accurate voltage clamp of cells with complex membrane geometry, such as photoreceptors, especially at higher current amplitudes. The present experiments used only a second-order approximation to the final nonlinearity, which limits its interpretation. Extension to higher order nonlinearities would be possible, but require much longer experiments to justify the increased number of fitting parameters.

While hypotheses of possible links between fitted parameters and physiochemical processes are interesting and may suggest further experiments, it must be emphasized that the present mathematical models were not designed to emulate specific biological mechanisms.

### Information transmission by photoreceptors

Linear information capacities of the experimental receptor current and receptor potential were lowest under the dimmest and brightest conditions (Figure 7). These results are not unexpected, because information capacity would be reduced by noise at the lowest light levels and by nonlinearity at the brightest levels. Similar maxima of information capacity at intermediate light intensities were found in the stick insect *Carausius morosus* (Frolov et al., 2012), the common backswimmer *N. glauca* (Immonen et al., 2014a), the water strider *Gerris lacustris* (Frolov and Weckström, 2014), and the lesser water boatman *Corixa punctata* (Frolov, 2015). This suggestion is also supported by the model values of information capacity. Since noise was absent from the models, information capacity was only limited by nonlinearity, which was maximal under the brightest conditions. Consequently, information was greatest at the lowest light intensity levels (Figure 7).

Entropy measurements can include both transduced signal and uncorrelated noise, but they are not dependent on linearity. If the models of receptor current and receptor potential are assumed to represent real photoreceptor behavior, the higher values of entropy seen in the experimental measurements than the model simulations (Figure 7) must represent contributions from uncorrelated noise. In this case, the additional noise in the real cells added about 20 Bits/s of entropy to the signal.

A nonlinear dynamic system does not necessarily lose information as long as the receiving system is designed to receive a distorted version of the input signal. However, a nonlinear system can easily lose information that can never be recovered at the output. A trivial example would be a squaring operation that produces a positive output for both positive and negative inputs, so that information about input sign is irretrievably lost. Interpreting the entropy data on this basis indicates that the model transmitted about 80% of the input signal entropy at low light levels, when it was behaving approximately linearly, but lost at least 50% of the input entropy when it became more nonlinear at high light levels. Inspection of the raw data confirms this interpretation (Figure 2). While the average amplitude of the naturalistic stimulus remained constant, the amplitude of the photoreceptor response dropped sharply during the first few seconds. This nonlinear change means that a receiver of the photoreceptor output could not reliably recover the absolute amplitude of the input signal. Information about the amplitude of input signal fluctuation was permanently lost.

## Conclusions

The three stage nonlinear model of phototransduction was able to predict receptor current and receptor potential output to naturalistic light fluctuations with reasonable fidelity. Importantly, the model could account for the strong change in response that occurs in the first seconds of stimulation to a dark adapted eye. Gain change probably occurs early in the process, possibly via screening pigment migration and feedback mechanisms such as Ca^2+^-dependent inhibition (Hardie and Minke, 1994; Song et al., 2012; Immonen et al., 2014a), and can be approximated by a simple exponential function of time. Other nonlinearities in the response are rapid, and probably include the effects of voltage activated ion channels. The dynamic properties of the main transduction machinery can be well-approximated by the log-normal model, but its basis remains unclear. While the nonlinear properties of photoreceptors cause a loss of information about the absolute level of light stimulation, the level of signal entropy transferred to the output suggests that estimates of information capacity are unrealistically pessimistic.

## Author contributions

EI and RF performed the animal experiments. ASF constructed the model and performed the data analysis. All authors contributed to designing the project and writing the paper.

### Conflict of interest statement

The authors declare that the research was conducted in the absence of any commercial or financial relationships that could be construed as a potential conflict of interest.
